# Transcriptome Profiles of *Sporisorium reilianum* during the Early Infection of Resistant and Susceptible Maize Isogenic Lines

**DOI:** 10.3390/jof7020150

**Published:** 2021-02-19

**Authors:** Boqi Zhang, Nan Zhang, Qianqian Zhang, Qianya Xu, Tao Zhong, Kaiyue Zhang, Mingliang Xu

**Affiliations:** 1 State Key Laboratory of Plant Physiology and Biochemistry, College of Agronomy and Biotechnology, National Maize Improvement Center, Center for Crop Functional Genomics and Molecular Breeding, China Agricultural University, 2 West Yuanmingyuan Road, Beijing 100193, China; bettyzhang1990@gmail.com (B.Z.); zhangnan@gdaas.cn (N.Z.); b20183020120@cau.edu.cn (Q.Z.); xuqy@cau.edu.cn (Q.X.); zhongtaofor@cau.edu.cn (T.Z.); zkaiyue1995@gamil.com (K.Z.); 2 Crops Research Institute, Guangdong Academy of Agricultural Sciences, Jinying 2 West Road, Tianhe District, Guangzhou 510640, China

**Keywords:** *Sporisorium reilianum*, *ZmWAK*, transcriptome

## Abstract

The biotrophic fungus *Sporisorium reilianum* causes destructive head smut disease in maize (*Zea mays* L.). To explore the pathogenicity arsenal of this fungus, we tracked its transcriptome changes during infection of the maize seedling mesocotyls of two near-isogenic lines, HZ4 and HZ4R, differing solely in the disease resistance gene *ZmWAK*. Parasitic growth of *S. reilianum* resulted in thousands of differentially expressed genes (DEGs) compared with growth in axenic culture. The protein synthesis and energy metabolism of *S. reilianum* were predominantly enriched with down-regulated DEGs, consistent with the arrested hyphal growth observed following colonization. Nutrition-related metabolic processes were enriched with both up- and down-regulated DEGs, which, together with activated transmembrane transport, reflected a potential transition in nutrition uptake of *S. reilianum* once it invaded maize. Notably, genes encoding secreted proteins of *S. reilianum* were mostly up-regulated during biotrophy. *ZmWAK*-mediated resistance to head smut disease reduced the number of DEGs of *S. reilianum*, particularly those related to the secretome. These observations deepen our understanding of the mechanisms underlying *S. reilianum* pathogenicity and *ZmWAK*-induced innate immunity.

## 1. Introduction

The soil-borne fungal pathogen *Sporisorium reilianum* causes head smut disease in maize (*Zea mays* L.), which poses a tremendous threat to global maize production [1,2]. The annual yield loss due to *S. reilianum* is generally around 6% but can reach 50% or more under severe infection [1,3,4]. The yeast-like haploid basidiospores of *S. reilianum* with compatible mating types recognize each other via the pheromone–receptor system and then conjugate to form pathogenic dikaryotic hyphae that invade maize roots or coleoptiles at the seedling stage [5]. Thereafter, the hyphae undergo a long endophytic growth phase to reach the florescence meristems, where they switch from vegetative to reproductive growth and produce large masses of spores in the ears and tassels [6]. *S. reilianum* also causes other symptoms, such as phyllody in the inflorescences, branching architectures, and a stunted phenotype [7,8,9]. Soil moisture and temperature are the crucial factors affecting smut incidence [8]. The optimal temperature for field infection is around 23−30 °C. Low soil water potential (−1.2 MPa) facilitates the convergence of compatible haploid strains, which may increase the disease severity [10]. 

The *S. reilianum* genome sequence has been published and shares a remarkably high degree of synteny with *Ustilago maydis* [11]. *S. reilianum* has a small genome of 18.7 Mb, comprising 23 chromosomes, to which 6648 gene models could be assigned after manual annotation [11]. The 467 *S. reilianum* proteins, characterized by secretion signal peptides and no transmembrane domains, are considered to be secreted effectors [12]. Among them, 256 possess known functions or domains, including 26 plant cell wall degrading enzymes [12]. The predicted secreted effectors are under high selective pressure, resulting from the biotrophic interaction of smut fungi with host plants [13]. So far, some virulence effectors have been identified in *S. reilianum*, and they play crucial roles in suppressing plant innate immunity. The symptom-affecting effector Sad1 causes suppression of apical dominance in *S. reilianum*–infected maize plants [14]. The virulence-affecting effector Pit2 could partially inhibit papain-like cysteine proteases essential for virulence [15,16]. The predicted cytoplasmic effector encoded by the *sr16441* gene was able to reliably suppress INF1-induced cell death [17]. At least three genes (*sr10057*, *sr10059*, and *sr10079*) at the cluster 19-1 of *S. reilianum* clearly contribute to disease incidence [18]. Many of these potential pathogenicity genes reside in clusters in the *S. reilianum* genome and are subject to more rapid evolution [11]. The availability of three smut genomes, i.e., of *Ustilago hordei*, *Ustilago maydis*, and *S. reilianum*, should facilitate the discovery of virulence effectors involved in the maize–*S. reilianum* pathosystem [11,19].

Maize resistance to head smut is a complex quantitative trait and varies considerably among different maize inbred lines and hybrids [8]. A number of quantitative trait loci (QTLs) have been identified using different mapping populations, including 11 QTLs mapped on eight chromosomes [20], five QTLs on four maize chromosomes [21], and one major QTL, *qHSR1*, on bin 2.09 [22]. A wall-associated kinase gene, *ZmWAK*, has been validated as the causal gene at *qHSR1* and reduces disease incidence by about 25% [23]. *ZmWAK* is highly expressed in the mesocotyls of maize seedlings, where it inhibits the growth of endophytic *S. reilianum*, resulting in fewer hyphae reaching the florescence meristems and thereby reducing disease incidence [23]. Introgression of *qHSR1* into 10 susceptible maize elite inbred lines via marker-assisted backcross selection enhanced disease resistance without changing other agronomic traits [24].

*S. reilianum* is being established as a model organism for studying host–fungus interaction. The availability of genome sequences of both *S. reilianum* and its host maize offers a unique opportunity to explore the molecular basis of the *S. reilianum*–host interaction, which is crucial to improving strategies to control severe head smut disease. Pathogenicity-related genes in smut fungi could be induced during fungal infection of a suitable host [25,26,27,28]. For instance, in-depth transcriptional profiling of *U. maydis* revealed numerous causal genes concerning fungal nutrition, defense suppression, and tumor induction during biotrophy [27].

Although *ZmWAK* is known to confer resistance to head smut, the molecular mechanisms by which *ZmWAK* influences the host–pathogen interaction are unclear. In the current study, we examined the transcriptome reprogramming of *S. reilianum* during its early infection of two near-isogenic lines with and without *ZmWAK*, which allowed us to detect the potential pathogenic effectors involved in the maize–*S. reilianum* interaction.

## 2. Materials and Methods

### 2.1. Axenic Culture Samples of S. reilianum for RNA Sequencing (RNA-seq)

Teliospores of *S. reilianum* were collected from the galls of infected maize (*Zea mays* L.) plants. The fungal galls were picked off, dried, and sifted. The resultant teliospores were used as the inoculum in the experiments. In preparation of axenic fungal samples, 100μL liquid potato dextrose agar (PDA) medium containing 10^3^/mL teliospores of *S. reilianum* were evenly inoculated on PDA (potato dextrose agar) plates and cultured under dark condition at 28 °C for 2 days. The hyphae were harvested by washing the plates with liquid PDA medium. The mixed hyphae were then inoculated in 50 mL PDA liquid medium at a volume ratio of 1:1000, and cultured at 28 °C on a rotary shaker at 200 rpm. When the optical density at 600 nm (OD_600_) reached 1.0, *S. reilianum* hyphae were harvested by centrifugation at 3000× *g* for 5 min, then directly frozen in liquid nitrogen. Samples were stored at −80 °C until RNA extraction. Two independent biological replicates were set up for the axenic culture sample of *S. reilianum*. Preparation of PDA medium: 200 g of sliced, peeled potatoes were boiled in 1 L of distilled water for 30 min. The potato infusion was filtered through cheesecloth. Then the infusion was mixed with 20 g dextrose (15 Agar for solid medium) and supplementary distilled water to 1 L and autoclaved for 15 min at 121 °C.

### 2.2. Infected Maize Mesocotyls for RNA-seq

A pair of maize near-isogenic lines (NILs), HZ4R and HZ4, were prepared as described by Zhang et al. [29]. HZ4R with *ZmWAK* displays resistance to head smut, while HZ4 without *ZmWAK* is susceptible to head smut. The two NILs show 99.65% identity in their genetic backgrounds [29]. The seeds were sterilized with 2% NaClO for 30 min and then washed three times with sterile double-distilled water. The culture substrate was prepared by mixing peat and vermiculite in a 1:1 ratio and autoclaved at 121 °C for 20 min. The sterilized seeds were sown in seedling trays with the sterilized culture substrate containing 0.1% teliospores (weight/weight). Plants were grown in an illumination incubator under 16-h-day/8-h-night conditions at 28 °C and 60% relative humidity. Apoptosis-like cell death started to manifest in the resistant NIL HZ4R at 7 days after sowing. At that time, infected mesocotyls were sampled, quick-frozen in liquid nitrogen, and stored at −80 °C until RNA extraction. For each of the resistant and susceptible NILs, we set up two biological replicates, each of which consisted of 20 mesocotyls.

### 2.3. Preparation of RNA-seq Libraries and Sequencing

Total RNA was extracted with TRIzol reagent (Invitrogen, Waltham, MA, USA) and purified with a PureLink RNA Mini Kit (Invitrogen). For preparation of the sequence library, RNA quality was subsequently assessed with an Agilent RNA 6000 Pico Kit. A cDNA library for each sample was constructed using an mRNA Seq sample prep Kit (Illumina, San Diego, CA, USA). Sequencing of each cDNA library was carried out on an Illumina HiSeq 2500 paired-end 100-bp (PE 100) system. The raw sequencing data were evaluated by FAST-QC.

### 2.4. Differential Gene Expression Analysis

We hypothesized that the *S. reilianum* genes involved in pathogenicity could be induced or switched on during its infection of maize. To capture these potential pathogenicity effectors, we generated RNA-seq data with deep coverage from the infected mesocotyls of HZ4 and HZ4R seedlings. We also performed RNA-seq on *S. reilianum* grown in axenic culture.

After filtration, the clean reads were aligned to the *S. reilianum* genome (version: *Sporisorium reilianum* f. sp. *zeae*) using Hisat2 software [30]. The relative abundance of mRNA was normalized and presented as fragments per kilobase per million mapped reads (FPKM). We applied the DEseq2 algorithm to process read counts and filter differentially expressed genes (DEGs) [31]. Genes whose relative mRNA abundance differed by at least 2-fold (fold change ≥2) with a false discovery rate (FDR) ≤ 0.05 were considered DEGs. We identified DEGs of *S. reilianum* by comparing the transcriptome profiles of *S. reilianum* between the axenic growth in vitro and parasitic growth in mesocotyls. The heatmaps for visualizing DEGs were generated by HemI software [32].

### 2.5. Transcript Annotation and Pathway Mapping

DEGs were annotated by the eukaryotic Clusters of Orthologous Groups (KOG) database to identify gene functions. The Fisher formula was used to examine the significance of KOG analysis. *p*-values < 0.05 indicate significant gene enrichment.

The *S. reilianum* genes were blasted onto the reference genome of *Saccharomyces cerevisiae*, and genes with correlation coefficient value < 0.00001 were reserved. Using Gene Ontology (GO), we classified gene functions into three categories: molecular function (MF), biological process (BP), and cellular component (CC). We used the Fisher formula to examine the *p*-value of each GO analysis, and *p*-values < 0.05 represent significant gene function [33,34,35].

The secretome of *S. reilianum* was identified using bioinformatic tools as described previously [36], and 467 *S. reilianum* proteins were considered to be secreted effectors. Within this secretome pool, we searched for the filtered DEGs, which were then considered to be secretome DEGs. Then we search the secretome DEGs locations on chromosome referring to EnsemblFungi website (http://fungi.ensembl.org/Sporisorium_reilianum/Info/Index, accessed on12 January 2021). The secretome DEGs distribution map was drawn by Mapchart [37].

## 3. Results

### 3.1. Global Gene Regulation of S. reilianum during Biotrophy

We obtained >165 million clean paired-end reads, which were then filtered and aligned to the *S. reilianum* genome. This resulted in >3.7 × 10^6^ reads of axenic-grown *S. reilianum* and ~6.3 × 10^5^ and ~5.7 × 10^5^ reads of *S. reilianum* grown in planta in HZ4 and HZ4R NILs, respectively (Table S1). The *S. reilianum* reads accounted for ~1.79% and ~1.57% of the total filtered reads of the HZ4 and HZ4R datasets, respectively (Table S1). Of the 6673 *S. reilianum* genes, approximately 77.2% and 69% were represented with >10 FPKM in the HZ4 and HZ4R datasets, respectively, and about 75.7% in the axenic dataset (Table S2), indicating that we effectively detected the expressed fungal genes of *S. reilianum* grown in either infected mesocotyls or axenic culture.

**Figure 1:**
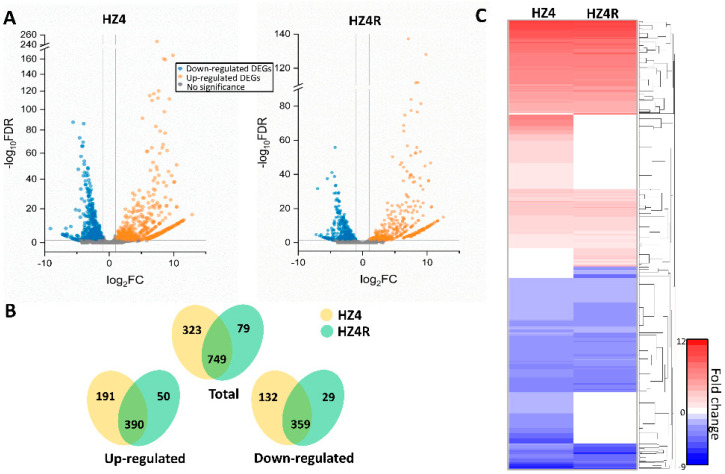
Differently expressed genes of *S. reilianum* grown in the infected mesocotyls and axenic culture. (**A**) The volcano map shows the comparison of gene expression levels between axenic and inoculated HZ4 (left) and HZ4R (right) near-isogenic lines (NILs). Blue dots represent genes that were significantly down-regulated; red dots represent genes that were significantly up-regulated; grey dots represent genes for which no significant differences were observed. (**B**) Venn diagrams show distinct and overlapping sets of differentially expressed genes (DEGs) in each group. All genes identified were differentially regulated by infection (fold change ≥ 2 and false discovery rate (FDR) ≤ 0.05) relative to control samples (axenic). (**C**) The heat map shows expression of DEGs of *S. reilianum*-infected HZ4 and HZ4R. Genes were clustered and log2 expression values are visualized relatively. Red indicates up-regulation, blue indicates down-regulation, and white indicates the background expression level for a gene. The data represent the average from two biological duplicates.

By comparing in planta growth with axenic growth, 1072 and 828 *S. reilianum* DEGs were identified in the infected mesocotyls of HZ4 and HZ4R, respectively (Figure 1A, Table S3). The number of *S. reilianum* DEGs was higher and their composition was more diverse in the HZ4 than the HZ4R dataset, demonstrating that a larger degree of transcriptome reprogramming occurred in *S. reilianum* after infecting the susceptible HZ4 NIL compared with the resistant HZ4R NIL.

In a Venn diagram analysis, we examined the shared or unique *S. reilianum* DEGs in HZ4 and HZ4R infected mesocotyls. Most *S. reilianum* DEGs (749) were common between the two NILs, and there were about four-fold more unique *S. reilianum* DEGs in HZ4 (323) than in HZ4R (79) (Figure 1B). In HZ4, uniquely, there were 191 up-regulated and 132 down-regulated *S. reilianum* DEGs, whereas in HZ4R, there were only 50 up-regulated and 29 down-regulated *S. reilianum* DEGs (Figure 1B). With respect to DEG function, 289 of the 359 common down-regulated *S. reilianum* DEGs have annotated functions, while only 151 of the 390 common up-regulated *S. reilianum* DEGs have annotated functions. For the unique *S. reilianum* DEGs, 225 of 323 in HZ4 have annotated functions, and 36 of 79 in HZ4R have annotated functions. The genes related to the secretome were observed in all DEG groups, but the frequencies varied greatly. Of the 390 common up-regulated DEGs, as many as 166 were annotated as secretome-related genes, while only 18 secretome-related genes were identified among the 359 common down-regulated DEGs. For the unique *S. reilianum* DEGs, 41 of 323 in HZ4 and 5 of 79 in HZ4R were secretome-related genes, respectively (Table S3). These findings suggest that different DEG groups consist of very different kinds of *S. reilianum* genes. 

Hierarchical clustering indicated that common *S. reilianum* DEGs, whether they were up- or down-regulated, showed similar expression patterns between the infected HZ4 and HZ4R mesocotyls (Figure 1C). The unique *S. reilianum* DEGs were clustered into four groups, corresponding to the up- and down-regulated DEGs specific to the HZ4 and HZ4R datasets, respectively (Figure 1C). Here, genes with unknown functions showed expression patterns similar to known genes, which helped us to infer the putative functions of these unknown genes and speculate their relevant metabolic pathways.

### 3.2. Functional KOG and GO Terms Enriched with DEGs

**Figure 2:**
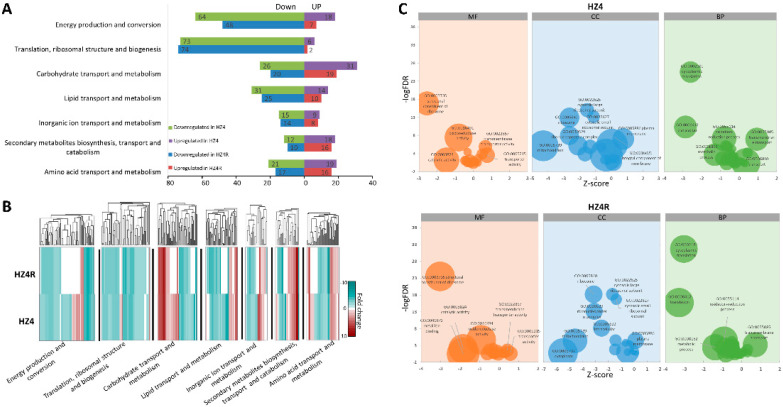
Functional annotation of DEGs. (**A**) The number of differentially expressed genes (DEGs) in Clusters of Orthologous Group (KOG)-based functional classification. The bar chart illustrates the number of DEGs significantly enriched and distributed among different KOG terms. (**B**) Gene expression pattern in different KOG terms. A heat map represents the hierarchical clustering of DEGs enriched in different KOG terms. Red indicates up-regulation, blue indicates down-regulation, and white indicates the background expression level for a gene. The data represent the average from two biological duplicates. (**C**) The distribution of DEGs in different GO terms. Bubble graphs demonstrate the significance of enrichment, gene numbers, and comparison of up-regulated and down-regulated gene numbers for a term. Bubble size presents gene number in a term; $Z-\mathrm{score}=\frac{\mathrm{up}-\mathrm{down}}{\sqrt{\mathrm{all}}}$.

Next, we annotated the *S. reilianum* DEGs by blast searches against the KOG database. The *S. reilianum* DEGs were significantly enriched in seven functional terms: (1) energy production and conversion (KOG:C); (2) translation, ribosomal structure, and biogenesis (KOG:J); (3) carbohydrate transport and metabolism (KOG:G); (4) lipid transport and metabolism (KOG:I); (5) inorganic ion transport and metabolism (KOG:P); (6) secondary metabolite biosynthesis, transport, and catabolism (KOG:Q); and (7) amino acid transport and metabolism (KOG:E) (Figure 2A). The up- and down-regulated *S. reilianum* DEGs of all functional KOG terms were higher in the HZ4 than in the HZ4R dataset, except for the down-regulated DEGs in the term KOG:J (translation, ribosomal structure and biogenesis) (Figure 2A). Most of the *S. reilianum* DEGs in the HZ4R dataset were included in the HZ4 dataset, and only a few were specific to the HZ4R dataset (Table S4). There were many more down-regulated DEGs than up-regulated DEGs in two KOG terms: KOG:C (energy production and conversion) and KOG:J (translation, ribosomal structure, and biogenesis) (Figure 2A,B). The massive number of down-regulated *S. reilianum* DEGs in these two development-related terms suggests that the growth of hyphae was arrested in the infected mesocotyls compared with the axenic culture. For four nutrition-related KOG terms, KOG:G (carbohydrate transport and metabolism), KOG:I (lipid transport and metabolism), KOG:P (inorganic ion transport and metabolism), KOG:Q (secondary metabolites biosynthesis, transport, and catabolism), and KOG:E (amino acid transport and metabolism), the numbers of up-regulated and down-regulated DEGs were roughly comparable (Figure 2A,B), indicating that nutrition uptake of *S. reilianum* was different before and after its infection of maize mesocotyls. In addition, the enrichment of DEGs in KOG:Q (secondary metabolites biosynthesis, transport, and catabolism) may reflect the strong physiological response of *S. reilianum* to its environmental conditions.

The *S. reilianum* transcripts were blasted to the available *Saccharomyces cerevisiae* database, resulting in 3615 significantly matched genes, which encompassed the 498 and 378 *S. reilianum* DEGs in the HZ4 and HZ4R datasets, respectively. GO analysis was conducted to assign the *S. reilianum* DEGs into three categories: MF, BP, and CC. The Z-score was adopted to represent differential numbers of the up- or down-regulated *S. reilianum* DEGs for a given GO term normalized to the standard deviation of the total DEG number. This evaluation allowed for a visual observation of the up- and down-regulated *S. reilianum* DEGs within a given functional term. The top GO terms with enriched *S. reilianum* DEGs were very similar between the HZ4 and HZ4R datasets (Figure 2C). Parallel comparison across the MF, BP, and CC categories revealed that the top GO terms were simply related to three important biological processes—protein synthesis, energy metabolism, and transmembrane transport—of which the first two were severely inhibited and the third was activated (Figure 2C). The translation-related GO terms in the MF (structural constituent of ribosome (GO:0003735)), BP (translation (GO:0006412), cytoplasmic translation (GO:0002181)), and CC (ribosome (GO:0005840), cytosolic large ribosomal subunit (GO:0022625), cytosolic small ribosomal subunit (GO:0022627), and ribonucleoprotein complex (GO:0030529)) categories were significantly enriched with down-regulated DEGs. Likewise, the energy-related GO terms in the MF (catalytic activity (GO:0003824), oxidoreductase activity (GO:0016491)), BP (oxidation–reduction process (GO:0055114), metabolic process (GO:0008152)), and CC (mitochondrion (GO:0005739)) categories were enriched with down-regulated DEGs. However, transmembrane transport-related GO terms in the MF (transmembrane transporter activity (GO:0022857), transporter activity (GO:0005215)), BP (transmembrane transport (GO:0055085), transport (GO:0006810)), and CC (plasma membrane (GO:0005886), integral component of membrane (GO:0016021)) categories were enriched with up-regulated DEGs. Only one GO term in the MF category, metal ion binding (GO:0046872), was unique to the HZ4R dataset enriched with 72 *S. reilianum* DEGs. Similar to the KOG analysis, after *S. reilianum* invaded the maize mesocotyl, reduced protein synthesis and energy metabolism affected the hyphal growth, while the enhanced transmembrane transport promoted the nutrition flow between the hyphae and mesocotyl.

### 3.3. The Secretome of S. reilianum Was Activated during Biotrophy

**Figure 3:**
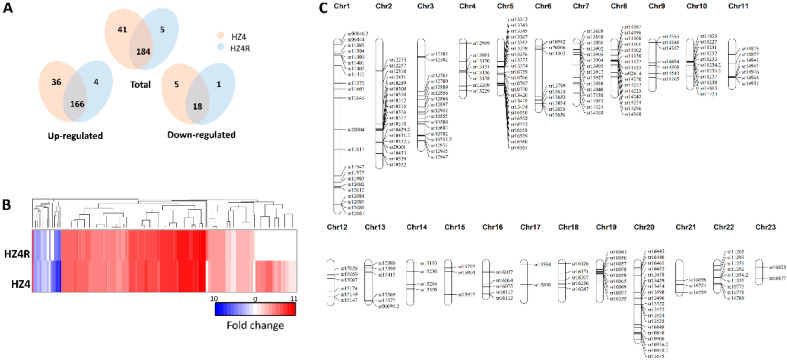
Secretome-related *S. reilianum* DEGs and their distribution on chromosomes. (**A**) Venn diagrams of differentially expressed genes (DEGs) encoding secreted proteins of *S. reilianum* after invasion of HZ4 and HZ4R. Venn diagrams show distinct and overlapping sets of DEGs in each group. All genes identified were differentially regulated by infection (fold change ≥ 2 and FDR ≤ 0.05) relative to control samples (axenic). (**B**) The heat map shows expression of DEGs encoding putative secreted proteins. Genes were clustered, and log2 expression values are visualized relative to the expression after infected maize. Red indicates up-regulation, blue indicates down-regulation, and white indicates the background expression level for a gene. The data represent the average from two biological duplicates. (**C**) DEGs encoding secreted proteins distributed on 23 chromosomes. Long bars represent 23 chromosomes, and black lines indicate the relative location of secretome DEGs.

The *S. reilianum* genome contains 467 genes encoding putative secreted proteins; among them, 211 have not yet been assigned functional annotations [12]. In the early infection of maize mesocotyls, 225 and 189 secretome-related *S. reilianum* genes were differentially expressed in the HZ4 and HZ4R NILs, respectively. Of them, 184 were shared by the two NILs, 41 were unique to HZ4, and 5 were unique to HZ4R (Figure 3A). More than half of the secretome-related DEGs were clustered in the *S. reilianum* genome on chromosomes 1-11, 19-20, and 22 (Figure 3C). About 90% of secretome-related *S. reilianum* DEGs were up-regulated, whereas only 45% of the remaining non-secretome-related DEGs were up-regulated in both HZ4 and HZ4R datasets (Figure 3A,B). With respect to the unique secretome-related DEGs, HZ4R has one down-regulated and four up-regulated DEGs, none of which had an annotated function by either GO or KOG analysis (Table S4). Among the 41 secretome-related DEGs unique to HZ4, the five down-regulated DEGs and 36 up-regulated DEGs had no functions annotated by GO analysis; however, 19 of them had annotated functions by KOG analysis, mainly related to KOG:G (5), KOG:R (5), and KOG:O (4) (Table S4). Of the 26 genes encoding carbohydrate-active enzymes, nine were consistently over-represented in the HZ4 and HZ4R datasets, of which eight were up-regulated and one was down-regulated (Table S5). The induction of carbohydrate-active enzymes is closely associated with pathogenicity and, thus, essential for the early infection of *S. reilianum* to both HZ4 and HZ4R lines.

Taken together, much more secretome-related genes were up-regulated than other *S. reilianum* genes during biotrophy, and *ZmWAK*-induced innate immunity may suppress a number of secretome-related genes required for fungal virulence to the susceptible HZ4 line.

## 4. Discussion

To reveal the pathogenicity arsenal of *S. reilianum*, we produced a deep-coverage transcriptome from infected mesocotyls at 7 days after sowing when discernible differences appeared between the susceptible and resistant NILs. Although the in planta fungal transcripts were relatively low in abundance, about 1.79% and 1.57% in the infected HZ4 and HZ4R mesocotyls, respectively, deep sequencing enabled us to capture most *S. reilianum* genes expressed in the infected mesocotyls, as 77.2% and 69% of the 6673 *S. reilianum* genes were represented with more than 10 FPKM in the HZ4 and HZ4R datasets, respectively (Table S2).

We identified *S. reilianum* DEGs separately in infected NILs with and without the resistance gene *ZmWAK* and then searched for shared and unique DEGs between the two NILs. While most *S. reilianum* DEGs were common between the HZ4 and HZ4R datasets, some were specific to one or the other. KOG analysis revealed a large number of down-regulated *S. reilianum* DEGs in two development-related terms, KOG:C and KOG:J. This was confirmed by GO analysis, in which the functional terms related to protein synthesis and energy metabolism were severely inhibited across all MF, BP, and CC categories. These results reveal that *S. reilianum* growth is vigorous in axenic culture but sluggish in infected mesocotyls.

Compared with HZ4R, more DEGs, whether they were up-regulated or down-regulated, were observed in the HZ4 dataset, indicating that more drastic transcriptome reprogramming occurred when *S. reilianum* invaded HZ4. KOG analysis revealed that the numbers of both up- and down-regulated *S. reilianum* DEGs were higher in the HZ4 than in the HZ4R dataset in almost all functional terms. Genetically, the resistance gene *ZmWAK* contributed to the difference in transcriptome reprogramming between the two NILs. As a pattern recognition receptor, ZmWAK may recognize and physically bind to the *S. reilianum* effector(s), triggering apoptosis-like cell death to confine *S. reilianum* hyphae into necrotic areas [29]. Therefore, ZmWAK would strengthen the innate immunity of HZ4R to diminish transcriptome reprogramming during *S. reilianum* infection.

Biotrophic plant–microbe interactions depend on the establishment of a nutritional interface through biotrophic hyphae, allowing nutrient flow between plants and microbial symbionts or pathogens [38]. KOG analysis indicated that *S. reilianum* DEGs were enriched in four nutrition-related terms, KOG:G, KOG:I, KOG:P, and KOG:E, with roughly comparable numbers of up-regulated and down-regulated DEGs. Similarly, GO analysis demonstrated that transmembrane transport-related terms were up-regulated in all MF, BP, and CC categories. Compared with its growth in axenic culture, *S. reilianum* would transit its nutrition uptake mode when infecting maize mesocotyls. As competitors of the host, endophytic hyphae pillage nutrients to facilitate exuberant fungal growth. Hexose transport-related genes (*Sr15905*, *Sr13579*, and *Sr16701*) were significantly up-regulated in the infected HZ4 and HZ4R mesocotyls, indicating that *S. reilianum* actively absorbed host sugar to support its parasitic growth (Table S3). Notably, the *S. reilianum* invasion also caused a transition in the energy supply of maize, from the tricarboxylic acid cycle to glycolysis [29]. By contrast, specific nutrient transition is not observed in *Colletotrichum graminicola*, the causal pathogen of anthracnose, during its infection of maize [39].

Filamentous pathogens such as smut fungi secrete proteins that act as modulators of host cell physiology and promote infection by reprogramming host cells [40]. Secreted proteins actively suppress plant immune responses, facilitate nutrition uptake, and regulate the infection process [12]. Many effectors contributing to pathogenicity are secreted proteins [28,41,42,43,44]. In our secretome analysis, about half of the 467 predicted secretory genes in the *S. reilianum* genome were differentially expressed in the early infection of maize mesocotyls, whereas the percentage of DEGs dropped to less than 15% for the remaining non-secretome-related *S. reilianum* genes (Tables S3 and S5). Moreover, about 90% of secreted DEGs were up-regulated, compared with ~45% of the remaining non-secretome-related *S. reilianum* genes. It is worth noting that the *S. reilianum* genes encoding the known pathogenicity-related secreted proteins were unanimously highly induced during biotrophy, for instance, *sr10077* and *sr10529* genes that encode the known virulence effectors Sad1 and Pit2 [14,15] and three secreted genes (*sr10057*, *sr10059*, and *sr10079*) that moderately promote *S. reilianum* virulence [18] (Table S3). It could be concluded that most secretome-related genes of *S. reilianum* were switched on or induced during biotrophy and those associated with pathogenicity then served as virulence effectors to facilitate hyphal colonization and expansion. 

Most *S. reilianum* DEGs are common in HZ4 and HZ4R, suggesting that they may play essential roles in invasion and expansion. For instance, nine carbohydrate-active enzymes that degrade the plant cell wall were consistently over-represented in the HZ4 and HZ4R datasets. Moreover, unique secreted proteins were much more abundant in the HZ4 (41) than in the HZ4R (5) datasets (Table S5). These findings suggest that *ZmWAK* could suppress some secreted DEGs required for *S. reilianum* to invade the susceptible HZ4 line, presumably achieved directly by binding secreted proteins or indirectly by inducing innate immunity.

## Data Availability

The data presented in this study are deposited in the National Center for Biotechnology Information (NCBI). The website link: https://www.ncbi.nlm.nih.gov/sra/PRJNA700975 (accessed on12 January 2021).

## Supplementary Materials

The following are available online at https://www.mdpi.com/2309-608X/7/2/150/s1, Table S1. Overview of reads mapping to S. reilianum and Z. mays; Table S2. FPKM of S. reilianum transcriptome; Table S3. Total DEGs; Table S4. KOG annotation of DEGs; Table S5. Secretome DEGs.
